# Early Identification of Isolated Sertoli Cell Dysfunction in Prepubertal and Transition Age: Is It Time?

**DOI:** 10.3390/jcm8050636

**Published:** 2019-05-09

**Authors:** Sandro La Vignera, Rosita A. Condorelli, Laura Cimino, Rossella Cannarella, Filippo Giacone, Aldo E. Calogero

**Affiliations:** Department of Clinical and Experimental Medicine, University of Catania, 95123 Catania, Italy; rositacondorelli@unict.it (R.A.C.); lauracimino@hotmail.it (L.C.); roxcannarella@gmail.com (R.C.); filippogiacone@yahoo.it (F.G.); acaloger@unict.it (A.E.C.)

**Keywords:** primary prevention, male infertility, male transition

## Abstract

The male transitional phase is of fundamental importance for future fertility. This aspect is largely neglected in clinical practice. This opinion aims to shed light on these issues. The children frequently complete the transition phase with a slight reduction of testicular volume. The system of detecting testicular volume is often inadequate. These patients evidently complete puberty in an incomplete way because they do not reach an adequate testicular volume, albeit in the presence of adequate height and regular secondary sexual characteristics.

Male infertility is constantly growing [1], with an estimated prevalence of approximately 15% of couples of childbearing age in industrialized countries [2]. The male factor, alone or in combination with the female one, contributes in about half of the cases of couple infertility [1]. Worryingly, meta-regression analysis recently showed a substantial decrease of sperm concentration and total count in the last 40 years, whose causes have not yet been identified [3]. Therefore, further studies aimed at understanding the etiology of such decline as well as the adoption of prevention strategies are urgently needed. 

The main interest has been mainly focused on secondary prevention strategies, mostly consisting in the treatment of the main diseases able to alter sperm quality (e.g., varicocele, urogenital infections, and endocrine disorders) [1,4,5]. Nevertheless, the evaluation of testicular function in prepubertal and transitional age would help in the early identification of testicular hypotrophy and signs of isolated tubulopathy and Sertoli cell (SC) dysfunction, thus timely recognizing a population at risk for male infertility. Accordingly, data from an epidemiological survey carried out in Italy by the Italian Society of Andrology and Medicine of Sexuality (SIAMS), revealed that 23% of 18 years old boys had low testicular volume (<12 mL) in a screening visit [6]. This evidence points to the importance of the adoption of measures aiming at evaluating testicular volume and function during the regular pediatric clinical practice. In this regard, the main concerns are when and how such investigation should be accomplished and which studies should be carried out to cover the limits of the current knowledge. 

To allow a timely identification of isolated tubulopathy and SC dysfunction, the investigation should start in the prepubertal age and the transition phase. The latter is the moment of transition from the pediatrician to the family doctor [7]. It usually occurs around the age of 14 and is characterized by the following steps: (1) Increase of testicular volume (>4 mL at orchidometry); (2) Tanner II pubic hair; (3) growth spurt; (4) presence of spermatozoa in first morning urine; (5) Tanner V pubic hair [7]. Commonly, the lack of growth spurt and of secondary sexual characters appearance (both signs of insufficient testosterone production) are easily detectable, the failure to achieve an adequate testicular volume, a parameter closely associated with the fertility potential [8], is not frequently reported, apart from patients with significant testicular hypotrophy [7]. However, its detection is important for the primary prevention of male infertility (Figure 1). 

The normal testicular volume in adulthood ranges between 12 and 25 mL [8]. In the clinical practice, a slight testicular volume decrease (e.g., 8–12 mL) of unknown origin occurs more frequently than severe testicular hypotrophy. Testicular volume measurement is usually done by orchidometry which overestimates it compared to ultrasound evaluation. Despite the latter is much more accurate, it cannot be proposed to all patients [7,9,10]. Therefore, an electronic calculator has recently been developed from the Research Institute at Nationwide Children’s Hospital to match ultrasound values. It requires the width of the testis, which may easily be obtained with a centimeter (cm) ruler at the physical examination, matched with the genital stage of development (G1 to G5) to elaborate a volume corresponding to each different phase of pubertal development [11]. Testicular volume nomogram and volume variations occurring in the transition through each stage of puberty in boys with normal and with delayed puberty have been described in a longitudinal study from a Danish cohort [12]. Testicular volumes expected according to the Tanner stage are reported in Table 1 [11].

Beyond the testicular volume, the evaluation of SC function deserves further attention. The hypothalamic-pituitary-gonadal axis is almost quiescent by definition in the prepubertal phase [13]. This make gonadotropins not representative of testicular dysfunction at this age. By contrast, gonadotropins are commonly adopted in the clinical practice to diagnose hypogonadism in adulthood. Therefore, additional markers are needed.

The quiescence of the hypothalamic-pituitary-gonadal axis before puberty does not implicate a lack of endocrine testicular function. Indeed, the testis is mainly made of immature SCs in childhood. These are nurse cells displaying a pivotal role in spermatogenesis. Accordingly, they provide functional support to germ cells, their nourishment and defense, through both SC-based blood-testis-barrier and the secretion of immunomodulatory factors. Before puberty, immature SCs actively proliferate and secrete large amounts of antimüllerian hormone (AMH), whose extent reflect the maturation degree of SCs. When puberty starts, SCs move from a proliferative and immature phase to a quiescent and mature one and start to express the androgen receptor. Concomitantly, AMH levels decrease. SCs secrete also inhibin B, whose production depends on follicle-stimulating hormone (FSH)-stimulation. Therefore, inhibin B serum levels physiologically increase after puberty [7]. 

Both AMH and inhibin B could be adopted for the evaluation of SC function in prepubertal and transitional phase. 

Low AMH levels in childhood reflect a SC dysfunction. Indeed, AMH levels depend on SC number and integrity. Low levels have been found in primary testicular disorders, such as cryptorchidism. In greater detail, low AMH levels have been found in 75% of children with bilateral cryptorchidism and nonpalpable testis and in 35% of those with inguinal testis [14]. Furthermore, the failure to have low AMH levels in the final stages of puberty expresses a condition of Sertolian functional immaturity, which possibly reflects decreased testosterone intratubular concentrations. The careful observation of the evolution of AMH levels in the course of pubertal development would allow us to receive the following information: (1) Adequate biological action of FSH; (2) adequate Sertolian proliferation; (3) appropriate expression of the androgen receptor by the SCs (functional maturity index); (4) adequate intratesticular biological action of testosterone [7]. These considerations highlight the importance of AMH measurement in prepubertal and transitional phase to timely identify any sign of SC dysfunction, responsible for primary testicular tubulopathy. 

Noteworthy are also the results coming from a study carried out on male patients with central hypogonadism, where FSH stimulation resulted in an increase of AMH levels [15]. These findings suggest that an increase of AMH levels after FSH administration occurs in the presence of normal SC function (which is typical in central hypogonadism). Therefore, a stimulation test (after standardization and identification of cut-off levels) might be proposed in childhood when SC dysfunction is suspected. 

The limits of AMH measurements should, however, be taken into account. Currently, it is performed by chemoluminescence. The specificity and sensitivity of the determination must be improved due to the absence of international reference standard, lack of comparability with the results of previous kit, and doubts about stability during the sample storage. Nonetheless, there are several evidences that allow us to know the normal values and the expected variations during puberty [16]. 

The significance of inhibin B levels have been also investigated in prepubertal and transition phase [17,18]. Low levels mainly reflect a defective FSH secretion and are useful in the differential diagnosis between congenital central hypogonadism and pubertal delay [19,20]. As far as its role in the early detection of a primary tubular dysfunction, low levels have been described in children with monolateral cryptorchidism compared to healthy ones [21,22], as well as in those with vanishing testis [22]. Therefore, they might represent a marker of SC integrity.

The possible role of insulin-like growth factor 1 (IGF1) for the achievement of testicular volume and function has been reported in experimental animals [23], but it requires further investigation in human being, especially in the light of the observed IGF1-induced SC proliferation in prepubertal animals [24].

Definitely, there is still the need to improve the reliability of testicular function parameters in prepubertal times, able to anticipate the diagnosis of testicular suffering, in particular the inadequate Sertolian function.

Further limits of the current practice deserving investigation regard the individuation of children at risk for the development of primary testicular tubulopathy. These include cryptorchidism, occurring in 2–4% of full-term and in 20–30% of premature births [25,26]; micropenis (which may hide partial androgen insensitivity syndrome even when present in isolated form) [27] and protracted hypoglycemia (pituitary disorders) [28]. Another less discussed topic concerns the possible cases of deficiency of minipuberty (physiological window of transient activation of the testicular pituitary gland in the neonatal period) characterized by increased levels of gonadotropins and testosterone [29]. The determination of INSL-3 (testicular hormone involved in the descent of the testicle in the fetal age) in children with cryptorchidism would allow us to have a marker that anticipates the decline of testosterone levels [30]. In addition, the offspring of mothers with gestational diabetes may be at risk for testicular tubulopathy predisposing to the development of cryptorchidism. Accordingly, a recent meta-analysis confirms an increased risk of cryptorchidism in births of mothers with gestational diabetes. The possible mechanism may be related to the decreased serum concentrations of sex hormone-binding globulin (SHBG) which cause the increase of free 17ß-estradiol and, in turn, undercut the production of INSL-3 [31]. 

More than 42 million overweight children were reported in 2014, with the prevalence doubling from 1980 to 2014 [32]. Whether obesity or hyperinsulinemia, both being widespread conditions in the pediatric population, especially in low- and middle-income countries [33], may affect SC function deserve to be investigated. 

Prepubertal male obesity in Tanner stage II already causes a decreased Leydig cell function [34]. Insulin has direct effects on spermatozoa where it finds adequate presence of glucose transporters and where it favors the availability of lactate within the SC for the production of energy. In particular, the expression of MCT4 (lactate transporter from the SC to the intratubular space for subsequent internalization within the germ cells) is under the control of insulin [35]. Insulin resistance is increased in patients with unexplained infertility [36,37]. Interestingly, inhibin B levels have been found to decline with increasing obesity in young men [38]. Furthermore, AMH and inhibin B levels have been found lower in obese adolescents with insulin resistance compared to normal weight controls. Therefore, obesity and insulin resistance may impact on SC function in prepubertal boys [39].

In summary, the evaluation of testicular volume and of markers of SC function in prepubertal and transitional age are of importance for the precocious identification of signs of primary testicular tubulopathy. A flowchart that may be used in the clinical practice is showed in Figure 2. Children at risk for testicular tubulopathy, including those with testicular hypotrophy, should undergo to AMH and inhibin B serum measurement. In case of normal values, the testicular volume should be measured at least every six months and should be framed in the context of the other auxological parameters till a sperm analysis can be requested (1.5 years after the onset of puberty [40]). In case of abnormal AMH or inhibin B values, a stimulation test with FSH might be proposed in the future after proper standardization [15]. The lack of response might represent indication for treatment with FSH since the vast majority of cases with testicular tubulopathy have FSH serum levels within the normal range [41].

## Figures and Tables

**Figure 1:**
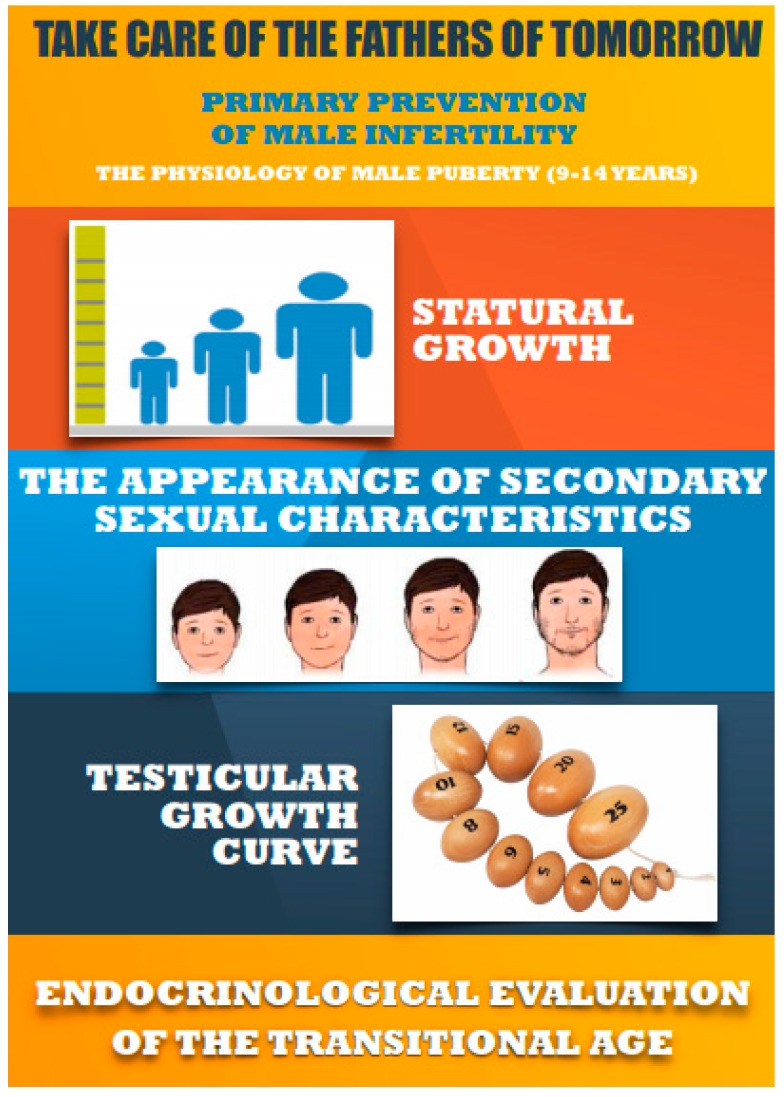
Primary prevention of male infertility. The importance of the testicular volume.

**Figure 2:**
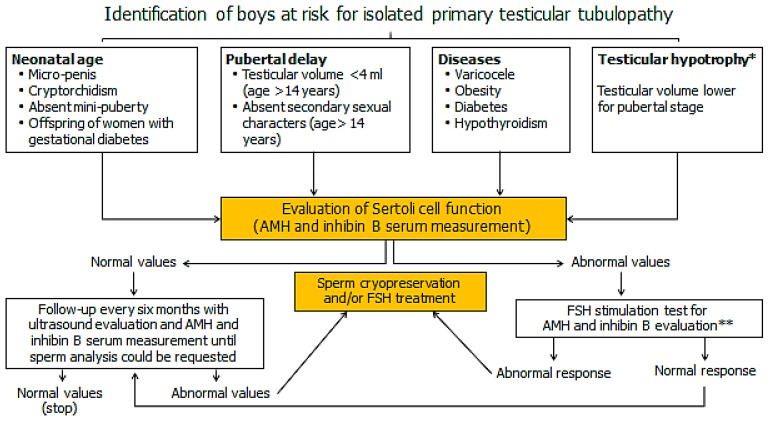
Flowchart proposed to help identify early testicular primary testicular tubulopathy in prepubertal and transitional age. * [42]; ** [15].

**Table 1:** Testicular volume values according to the Tanner stage. Legend: The testicular volume is reported as median value [11].

Tanner Stage	Testicular Volume (mL)
I	0.71
II	3.62
III	6.42
IV	10.85
V	17.32
